# Trends, Predictors, and Outcomes of Critically Ill Patients With Sickle Cell Disease in the United States

**DOI:** 10.1002/jha2.70111

**Published:** 2025-08-13

**Authors:** Tochukwu Nzeako, Olayemi Adeniran, Saranjeet Kaur, Shreya Singh, Jen Wang, Ammar Alzoubi

**Affiliations:** ^1^ Department of Internal Medicine Christiana Care Hospital Newark Delaware USA; ^2^ Department of Cardiovascular Diseases SUNY Downstate Health Sciences University Brooklyn New York USA; ^3^ Department of Epidemiology West Virginia University Morgantown West Virginia USA; ^4^ Department of Medicine Touro College of Osteopathic Medicine Manhattan New York USA; ^5^ Department of Hematology and Oncology Brookdale University Hospital Medical Center Brooklyn New York USA; ^6^ Department of Hematology and Oncology Christiana Care Hospital Newark Delaware USA

**Keywords:** adverse clinical events, critically ill, intensive care unit, sickle cell disease

## Abstract

**Introduction:**

Despite recent advances in sickle cell disease (SCD) research and management, there remains very limited information available on critically ill SCD patients requiring intensive care units (ICUs).

**Methods:**

The National Inpatient Sample was queried using the International Classification of Diseases codes to identify critically ill patients with SCD requiring ICU admission. These patients were further stratified into those with and without adverse clinical events (ACEs). The study outcomes were the incidence of acute chest syndrome, cardiac arrest, hemodialysis, sepsis, shock, and transfusion.

**Results:**

Of 5941 patients admitted to the ICU, 2826 (47.6%) had ACE. Patients with ACE were likely to be older, male, white, rural, and have higher comorbidities. The prevalence of ICU admission increased by 126% (*p* < 0.01). The predictors of ACE included male sex, older age, coronary artery disease, heart failure, renal failure, and two or more comorbidities. Patients with ACE were more likely to have cardiac arrest, hemodialysis, sepsis, and shock (all *p* < 0.01).

**Conclusion:**

There has been an increase in the prevalence of critically ill SCD patients requiring ICU care, with subsequent morbidity and mortality. Further research is needed to understand the underlying factors that drive these observed trends and increase mortality rates.

**Trial Registration:**

The authors have confirmed clinical trial registration is not needed for this submission.

## 1 Introduction

Sickle cell disease (SCD) is a genetic red blood cell disorder caused by a mutation in the beta‐globin chain of hemoglobin molecules, resulting in an abnormal type of hemoglobin, hemoglobin S (Hb S) [1]. In the United States, SCD has a prevalence of over 100,000, many of whom are African Americans [2, 3]. SCD is characterized by the polymerization of sickle‐shaped hemoglobin within the red blood cells, causing deformation and rigidity of the cells, which obstruct blood vessels, resulting in vaso‐occlusive events [4]. The clinical picture of SCD is dominated by complications arising from these vaso‐occlusive events, including anemia, acute chest syndrome (ACS), stroke, and kidney injury [5, 6].

Individuals with SCD are vulnerable to frequent hospitalizations and eventual intensive care unit (ICU) admissions due to the complications [7, 8]. Furthermore, the risk of worse outcomes among those associated with ICU admissions remains high [9]. The reasons for ICU admissions in SCD patients are usually heterogeneous and may be from SCD‐related cases or other events. Reported studies have identified that ACS constitutes most ICU admissions among SCD patients, with associated substantial mortality rates [10, 11]. Although these risks are well described, data on the characterization of ICU admission and associated outcomes among SCD patients in the United States are lacking. Thus, it is imperative to evaluate the possible risk factors for ICU admissions and complications to implement preventive strategies aimed at reducing the mortality rate.

Therefore, the study aimed to examine the trends, outcomes, and predictors of ICU admissions among SCD patients using nationally representative hospitalization data in the United States.

## 2 Methods

### 2.1 Data Source

The study utilized the National Inpatient Sample (NIS), which is part of the Healthcare Cost and Utilization Project (HCUP), sponsored by the Agency for Healthcare Research and Quality (AHRQ) [12]. The NIS is the largest all‐payer‐inpatient database derived from community hospitals in all participating states, representing more than 97% of the US population. The database is a stratified sample of annual discharge records of approximately eight million hospital stays. Each discharge record contained diagnoses and procedures coded using the International Classification of Diseases, Tenth Revision (ICD‐10) codes. Institutional Review Board approval was not required for this study, given that the database was de‐identified and patient confidentiality was protected.

### 2.2 Study Population and Variables

**FIGURE 1:**
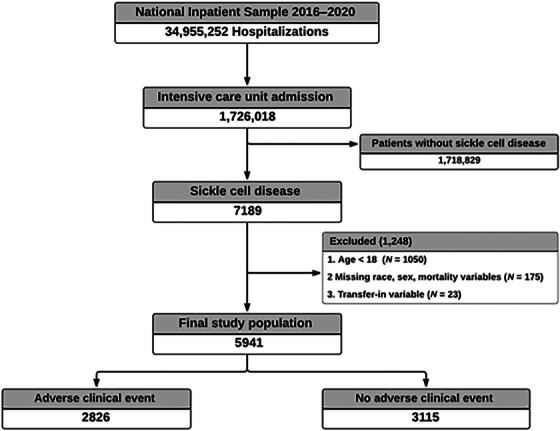
Decision flowchart of eligible records identified using the National Inpatient Sample (NIS) showing the selection process and final study population.

The NIS database was analyzed to identify US adults aged 18 and older with primary and secondary hospitalizations for SCD and ICU admissions between January 1, 2016 and December 31, 2020. First, we queried the database to identify all the ICU admissions. The database does not contain a variable that describes ICU admissions. Therefore, we defined ICU admissions as patients with all hospitalizations requiring mechanical ventilation and vasopressors, as identified using ICD‐10 procedure codes, as described in other studies [12, 13, 14]. Second, we selected patients with SCD from the overall ICU admissions. SCD was identified using ICD‐10 codes D57x, Hemoglobin SS (HbSS), Hemoglobin C (HbC), sickle cell thalassemia, and other sickle cell types. These patients were further stratified into those with and without adverse clinical events (ACEs). ACEs were defined as a composite of death and the use of life‐sustaining interventions, such as extracorporeal membrane oxygenation (ECMO). The selection process is represented in Figure 1.

Furthermore, we extracted patient characteristics, such as age, sex (male and female), race/ethnicity (Whites, Blacks, Hispanics, Asians or Pacific Islanders, and Native Americans and others), insurance status (Medicare, Medicaid, Private, self‐pay, and others), and comorbidities (hypertension, diabetes mellitus, heart failure, coronary artery disease, renal failure, liver failure, chronic lung disease, peripheral vascular disease, cancer, valvular heart disease, coagulopathy, paralysis, and obesity). We compared the differences in characteristics between the two study groups (ACE and non‐ACE), including age, sex, race, payer, and comorbidities. A summary of the ICD‐10 codes used to identify these comorbidities is presented in Figure S1.

### 2.3 Outcome Measures

The study outcomes included ACS, cardiac arrest, hemodialysis, sepsis, shock, and transfusion. In addition, we investigated the prevalence trends of ICU admission in SCD patients. All outcomes were selected based on the existing literature on SCD. Supporting Information includes a list of ICD‐10 diagnoses and procedure codes for identifying these outcomes (see Table S1).

### 2.4 Statistical Analysis

Weighted estimates were generated by applying survey trend weights to account for data stratification as recommended by the AHRQ. Continuous variables are presented as mean and standard deviation (SD), and categorical variables are presented as percentages. Baseline characteristics were compared using an independent sample *t*‐test for continuous variables and the chi‐square and Fisher's exact tests for categorical variables. We calculated trends using the weighted estimates of ICU hospitalizations in SCD patients divided by the total number of hospitalizations in a given year per 100,000. In addition, trends in outcomes were derived as the total number of events per 100 hospitalizations. Trend analyses were performed using the Cochran–Armitage trend test.

We assessed potential predictors of ACE using a backward selection multivariable logistic regression model adjusted for age, sex, race or ethnicity, location, insurance status, income, hospital region, hypertension, diabetes, anemia, heart failure, coronary artery disease, lung diseases, liver failure, renal failure, cancers, obesity, smoking, stroke, and comorbidity index. The missing values in the dataset constituted 2.5% and were removed from the overall study population. A two‐tailed *p* < 0.05 was considered statistically significant. Data manipulation and statistical analyses were performed using the SAS software (version 9.4; SAS Institute Inc., Cary, NC, USA).

## 3 Results

### 3.1 Characteristics of the Study Population

During the study period, we identified 5941 SCD hospitalizations that required ICU admissions, of which SCD patients accounted for 0.03% of the total ICU admissions and 3.1% of the overall sickle cell patient population. The baseline characteristics of the overall SCD population requiring ICU admissions, stratified by the presence or absence of ACEs, are detailed in Table 1. Of the included patients, 2826 (47.6%) had ACE. Patients with ACE had a higher mean age of 57.0 years compared with 46.7 years for patients without ACE. Patients with ACE were likely to be male, white, rural, or of the HbSS genotype. They had more comorbidities, including anemia, cancer, coagulation deficiency, coronary artery disease, diabetes, heart failure, hypertension, liver failure, obesity, renal failure, and stroke. The reasons for admission to the ICU were hematologic (directly related to SCD complications) and non‐hematologic (Table 2). Non‐hematologic causes were the most common reasons for ICU admission (63.6%) compared to hematologic causes (36.4%). Sepsis was the most common reason for admission, accounting for 23.6%. ACS constituted 3.3% of ICU admissions, followed by vaso‐occlusive crisis (VOC) (5.2%), stroke (1.6%), anemia (1.2%), and pulmonary embolism (0.7%) (Table 2).

**TABLE 1:** Baseline characteristics of critically ill patients with sickle cell disease in the United States, 2016–2020.

Characteristics	Total	ACE	No‐ACE	
Unweighted (Weighted)	5941 (29,705)	2826 (14,130)	3115 (15,575)	*p* value
**Age, mean (SD)**	51.6 (18.6)	57.0 (17.0)	46.7 (18.7)	< 0.0001
				
18–39	31.0	18.5	42.3	
40–59	30.7	32.7	28.8	
≥ 60	38.3	48.7	28.9	
				
**Sex**				< 0.0001
Male	42.1	47.7	37.1	
Female	57.9	52.3	62.9	
				
**Race**				< 0.0001
White	25.1	32.5	18.3	
Black	66.1	58.6	72.9	
Hispanics	5.1	5.1	5.1	
Asians	0.9	1.1	0.8	
Others	2.8	2.8	2.8	
				
**Payer**				< 0.0001
Medicare	47.5	57.8	38.1	
Medicaid	27.1	19.9	33.6	
Private	19.1	16.4	21.5	
Self‐pay	3.3	2.9	3.6	
Others	3.1	3.0	3.2	
				
**Income**				0.0282
$1–49,999	43.3	41.2	45.3	
$50,000–64,999	23.3	24.6	22.1	
$65,000–85,999	18.0	18.4	17.7	
≥ $86,000	14.1	14.4	13.8	
Missing	1.3	1.4	1.1	
				
**Hosp. region**				
Northeast	19.8	16.1	23.1	
Midwest	25.1	27.4	23.0	
South	47.1	48.8	45.6	
West	8.1	7.7	8.4	
				
**Location**				0.0007
Rural	8.7	10.1	7.5	
Urban	91.3	89.9	92.5	
				
**Genotype**				
HbSS	50.6	61.5	40.7	< 0.0001
HbC	36.2	48.2	25.4	< 0.0001
Sickle cell thalassemia	36.2	47.5	25.9	< 0.0001
Others sickle cell type	34.5	46.3	23.8	< 0.0001
				
**Comorbidities**				
Anemia	53.8	65.8	42.8	< 0.0001
Cancer	10.7	12.3	9.3	0.0002
Coagulation deficiency	35.9	46.5	26.4	< 0.0001
Coronary artery disease	21.8	29.9	14.4	< 0.0001
Diabetes	34.9	42.2	28.3	< 0.0001
Heart failure	45.2	58.6	33.0	<.0001
Hypertension	63.5	74.8	53.3	<.0001
Liver failure	26.0	33.1	19.6	<.0001
Lung disease	30.1	30.1	30.0	0.9339
Obesity	27.5	29.0	26.1	0.0164
Renal failure	44.1	59.9	29.8	< 0.0001
Smoking	16.0	14.8	17.2	0.0126
Stroke	22.9	28.1	18.2	< 0.0001
				
**Elixhauser comorbidity**				< 0.0001
0	8.5	2.3	14.2	
1	10.2	4.4	15.4	
2	9.9	7.3	12.2	
≥ 3	71.4	86.0	58.2	

**TABLE 2:** Primary reasons for admissions in critically ill patients with sickle cell disease, stratified by hematologic and non‐hematologic causes.

Total ICU admission	Patients, *n*	Percentage, %
**Hematologic**	**2164**	**36.4**
Acute chest syndrome	197	3.3
Vaso‐occlusive crisis	307	5.2
Anemia	71	1.2
Pulmonary embolism	44	0.7
Stroke	96	1.6
Sepsis	1400	23.6
Other hematologic complications	49	0.8
**Non‐hematologic**	**3777**	**63.6**
Cardiovascular diseases	641	10.8
Endocrine diseases	105	1.8
Digestive diseases	359	6.0
Genitourinary diseases	123	2.1
Neoplasms	204	3.4
Neurology	262	4.4
Pneumology	550	9.3
Rheumatology	85	1.4
Others	1448	24.4

### 3.2 Trends for Prevalence of ICU Admission in SCD

Between 2016 and 2019, the prevalence of ICU admissions among SCD patients increased from 12.7 per 100,000 in 2016 to 28.8 per 100,000 in 2020 (*p* = < 0.01), as shown in Table 3. This increase in overall ICU admission was reflected in increases across all age groups, sexes, and racial/ethnic groups (Table 3). In contrast, no significant difference was found in ACE trends (*p* = 0.15). Moreover, during the same period, the mean length of hospital stays increased from 15 ± 6 to 17 ± 20 days (*p* = 0.02), and the cost of hospitalization increased from 47,892 ± 50,603 to 65,400 ±77,478 days (*p* = 0.01) (Table 3).

**TABLE 3:** Trends in intensive care admissions among patients with sickle cell disease, 2016–2020.

Characteristics	Total *N* = 29,705	2016 *N* = 3650	2017 *N* = 5305	2018 *N* = 6315	2019 *N* = 6725	2020 *N* = 7710	*p* value for trend
**SCD intensive care** **per 100,000**	20.7	12.7	18.2	21.5	22.9	28.8	0.00
**Age, mean (SD)**	51.6 (18.6)	47.0 (17.3)	51.6 (18.8)	52.2 (18.8)	53.0 (18.8)	52.3 (18.3)	0.11
**Age**							
18–39	26.9	20.9	24.3	27.0	28.5	34.5	0.00
40–59	27.0	17.0	23.1	27.6	29.1	39.9	0.01
≥ 60	15.1	6.8	13.1	16.4	17.8	21.3	0.01
							
**Sex**							
Male	20.4	12.5	19.4	21.1	22.5	26.8	0.01
Female	20.9	12.9	17.2	21.9	23.2	30.3	0.00
							
**Race**							
White	7.7	2.4	7.3	9.1	8.9	11.4	0.03
Black	89.4	66.0	77.5	90.5	98.0	115.6	0.00
Hispanics	9.4	5.8	6.9	9.9	9.4	14.8	0.02
Asians	7.0	2.6	6.2	7.3	8.5	10.5	0.01
Others	15.8	12.3	13.5	11.6	18.0	24.1	0.07
**Outcomes**							
Adverse clinical events	47.6	37.9	48.7	48.7	48.2	49.9	0.15
Acute chest syndrome	40.9	20.1	41.8	42.9	43.9	45.8	0.11
Cardiac arrest	15.1	9.9	18.0	15.0	15.0	15.7	0.44
Hemodialysis	11.7	9.2	11.4	12.6	12.8	11.5	0.23
Sepsis	41.7	34.5	42.6	42.5	40.1	45.3	0.15
Shock	12.4	5.8	10.3	13.2	15.4	13.6	0.05
Transfusion	17.2	22.5	18.6	17.3	16.4	14.5	0.01
**Resource utilization**							
Length of stay, mean (SD) days	16.7 (20.1)	15.0 (16.2)	16.4 (18.0)	16.5 (19.8)	17.2 (23.5)	17.3 (20.2)	0.02
Cost of hosp., mean (SD) $	58,504 (74,824)	47,892 (50,603)	54,966 (60,118)	54,536 (67,942)	62,699 (95,266)	65,400 (77,478)	0.01

*Note*: The overall study population was stratified according to the presence of adverse clinical events. The variables were represented as percentages. The age variable was represented as both continuous (mean and standard deviation) and categorical (percentages).

Abbreviations: hosp., hospitalization; SCD, sickle cell disease; SD, standard deviation.

### 3.3 Predictors of Adverse Clinical Events

After adjustment for age, sex, race/ethnicity, insurance, hospital region, income, location, and comorbidities, male sex (OR, 1.13 [95% CI, 1.01–1.27], *p* = 0.04), increasing age groups: 40–59 years (OR, 1.40 [95% CI, 1.19–1.65], *p* = 0.02) and ≥ 60 years (OR, 1.46 [95% CI, 1.22–1.75], *p* < 0.00), HbSS genotype (OR, 1.59 [95% CI, 1.35–1.87], *p* < 0.01) or HbC genotype (OR, 1.76 [95% CI, 1.24–2.48], *p* < 0.01), coronary artery disease (OR, 1.35 [95% CI, 1.16–1.58], *p* < 0.01), heart failure (OR, 1.21 [95% CI, 1.05–1.39], *p* = 0.01), renal failure (OR, 1.88 [95% CI, 1.64–2.14], *p* < 0.01), and increasing number of Elixhauser comorbidity index were independently associated with higher odds (Elixhauser = 1: OR, 1.73 [95% CI, 1.24–2.41], *p* < 0.01); (Elixhauser = 2: OR, 3.29 [95% CI, 2.38–4.54], *p* < 0.01); (Elixhauser ≥ 3: OR, 4.49 [95% CI, 3.28–6.16], *p* < 0.01) of ACEs among SCD patients admitted to ICU (Table 4).

**TABLE 4:** Predictors of adverse clinical events (ACEs) in critically ill patients with sickle cell disease.

Variables	Adjusted OR	95% CI	*p* value
Age group			
18–39	Ref	Ref	Ref
40–59	1.40	1.19–1.65	0.0189
≥ 60	1.46	1.22–1.75	0.0033
Male	1.13	1.01–1.27	0.0359
Genotype			
HbSS	1.59	1.35–1.87	< 0.0001
HbC	1.76	1.24–2.48	0.0014
Others SC	0.61	0.41–0.89	0.0111
Coronary artery disease	1.35	1.16–1.58	< 0.0001
Heart failure	1.21	1.05–1.39	0.0066
Hypertension	0.87	0.75–1.02	0.0858
Lung disease	0.73	0.64–0.83	< 0.0001
Obesity	0.79	0.70–0.90	0.0004
Renal failure	1.88	1.64–2.14	< 0.0001
Smoking	0.70	0.60 ‐ 0.82	< 0.0001
Elixhauser comorbidity			
0	Ref	Ref	Ref
1	1.73	1.24–2.41	0.0031
2	3.29	2.38–4.54	< 0.0001
≥ 3	4.49	3.28–6.16	< 0.0001

### 3.4 In‐Hospital Outcomes

Patients with ACE were more likely to have had cardiac arrest (OR, 1.35 [95% CI, 1.16–1.58], *p* < 0.01), hemodialysis (OR, 1.35 [95% CI, 1.16–1.58], *p* < 0.01), sepsis (OR, 1.35 [95% CI, 1.16–1.58], *p* < 0.01), and shock (OR, 1.35 [95% CI, 1.16–1.58], *p* < 0.01). Conversely, there were no differences in ACS or transfusion between the two groups (Table 5).

**TABLE 5:** Adjusted odd ratios of outcomes of critically ill patients with sickle cell disease.

Outcomes	Adjusted OR	95% CI	*p* value
Acute chest syndrome	0.87	0.67–1.13	0.2915
Cardiac arrest	3.56	2.98–4.24	< 0.0001
Hemodialysis	1.42	1.18–1.71	0.0002
Sepsis	1.42	1.26–1.60	< 0.0001
Shock	1.73	1.44–2.07	< 0.0001
Transfusion (blood/exchange)	0.96	0.82–1.11	0.5680

## 4 Discussion

In this large population‐based study, we investigated the trends, predictors, and outcomes in critically ill adults with SCD. The significant findings of this study were as follows: (1) an increase in the trends of critically ill SCD patients requiring intensive care management. We found an increase in trends of ICU admission across all age groups, sexes, and races/ethnicities; (2) predictors of ACEs among critically ill SCD patients admitted to the ICU included increasing age, male sex, presence of heart failure and renal failure, and an increasing number of comorbidities; and (3) patients with ACEs were likely to have cardiac arrest, hemodialysis, sepsis, and shock. Our study is the first and largest national study to examine critically ill adults with SCD in the United States.

Improvements in the management of SCD in the last few decades have led to increased survival among children and a subsequent increase in the proportion of adults with SCD who reach adulthood [15, 16]. The course of SCD in the adult population varies from benign to severe, requiring ICU management [17]. In our study, the prevalence of critically ill patients with SCD requiring ICU admission increased by approximately 126% during the study period. This increase was further reflected across all age groups, sexes, and racial and ethnic groups. Prior studies have not examined trends in ICU admissions in patients with SCD. However, a US study that investigated hospitalizations and readmissions among SCD patients highlighted age as the most significant predictor of hospitalizations and readmissions [18]. This is consistent with the results of our study, which found an increase in ICU admissions with increasing age. Patients aged 60 years and older had an approximately 213% increase compared to patients 40–59 years and 18–39 years (135% and 65%, respectively).

Previous studies have reported ACS as the most common reason for ICU admission in SCD patients [19, 20, 21]. This contrasts with our study, which found that sepsis was the most common primary reason for admission, accounting for 23.6% of all ICU admissions. VOC and ACS constituted 5.2% and 3.3% of all the primary reasons for admission, respectively. The variation in our study from prior studies might be explained by differences in the databases used for the analysis. Moreover, the diagnosis of ACS is underestimated, as most patients are initially diagnosed with acute respiratory failure before further management of ACS [11]. In addition, studies have reported that ACS may develop during hospitalization after the initial presentation of a painful VOC or sepsis [22, 23].

In the present study, approximately 48% of critically ill SCD patients admitted to the ICU had ACEs, a composite of death and the use of life‐sustaining interventions, such as ECMO. Patients with ACEs were likely to be older, in the lowest quartile of income, and have many comorbidities. In addition, in‐hospital mortality alone accounts for 22% of our study population. Our study supports prior international studies' findings that found the in‐hospital mortality in SCD patients admitted to the ICU to be about 12%–22% [10, 20, 21]. Also, a retrospective study in France estimated ICU mortality to be 3.3% [24]. Many of these studies range from single to tertiary centers and consist of small sample sizes. Several predictors of mortality of SCD patients in the ICU included older age, use of mechanical ventilation, and renal replacement therapy. Our results found that increasing age, male sex, and the presence of heart failure or renal failure with two or more comorbidities are good predictors of ACEs. In addition, patients with ACEs were likely to experience cardiac arrest, hemodialysis, sepsis, and shock.

This study utilized a nationally representative database to investigate SCD patients admitted to the ICU. The strengths of this study are the large sample size of patients with SCD and its population‐based design. However, the findings of this study should be cautiously interpreted, considering the following limitations. First, the NIS database is an administrative database based on ICD‐10 coding, which can lead to the misclassification of cases. Our study identified the SCD population using the ICD‐10 code D571, susceptible to coding errors. However, these codes have been validated in prior studies to identify most SCD patients with a high positive predictive value [25]. In addition, critically ill patients were selected based on the procedure codes for the use of vasopressors and/or mechanical ventilation. While these procedure codes have been applied in multiple studies to identify patients requiring ICU admission, SCD patients could still be admitted to the ICU without these two requirements, thereby leading to an underestimation of the correct prevalence of critically ill SCD patients. Second, the NIS does not identify individual patients, and recurrent hospitalization episodes for each patient are coded as separate observations, which could lead to an overestimation of cases. Third, due to the retrospective nature of the database, there remains a potential for bias due to confounding factors despite adjustment for comorbidities. Despite these limitations, our study provides valuable and robust information on critically ill SCD patients, an area currently lacking in the United States. Future analyses should involve each subgroup whenever possible to improve our understanding of this field. Addressing these concerns is essential for future studies.

## 5 Conclusion

In summary, this study revealed that the prevalence rate of SCD patients requiring ICU care is increasing. In addition, ICU admission was associated with higher adverse clinical outcomes, including in‐hospital mortality and the use of life‐sustaining machines, such as ECMO. This increase in prevalence is associated with higher ICU stays and increasing hospitalization costs, thus constituting a substantial health burden to the United States. However, further research is needed to fully understand the underlying factors driving these observed trends and the increased mortality.

## Author Contributions

Tochukwu Nzeako, Olayemi Adeniran, Saranjeet Kaur, Shreya Singh, Jen Wang, and Ammar Alzoubi contributed to the drafting, original draft preparation, methodology, and final review of the manuscript. Jen Wang and Ammar Alzoubi supervised the study.

## Ethics Statement

This study did not involve primary data collection from human participants, as it exclusively utilized secondary data from publicly available sources, specifically the National Inpatient Sample database, which is de‐identified and exempt from requiring ethical approval. All research activities were conducted in compliance with relevant ethical guidelines, including the Declaration of Helsinki.

## Consent

The authors have nothing to report.

## Conflicts of Interest

The authors declare no conflicts of interest.

## Supporting information




**Figure S1**: *ICD‐10* Codes Used to Identify Independent and Dependent Variables. **Table S1**: International Classification of Disease 10th Revision (ICD‐10) codes for comorbidities

## Data Availability

The datasets used and/or analyzed during the current study are available from the corresponding author on reasonable request.
