# Supplementary material for: Trends, Predictors, and Outcomes of Critically Ill Patients With Sickle Cell Disease in the United States
Source: EJHaem. 2025 Aug 13;6(4):e70111. doi: 10.1002/jha2.70111 (PMC12345595; doi:10.1002/jha2.70111)

***Submission to the EJHAEM***

**Title: Trends, Predictors, and Outcomes of Critically Ill Patients with Sickle Cell Disease in the United States.**

**Supplementary Tables and Figures**

**FIGURE LEGENDS**.

**Supplementary Figure 1.** *ICD-10* Codes Used to Identify Independent and Dependent Variables

S1 Table. *International Classification of Disease 10^th^ Revision* (ICD-10) codes for comorbidities


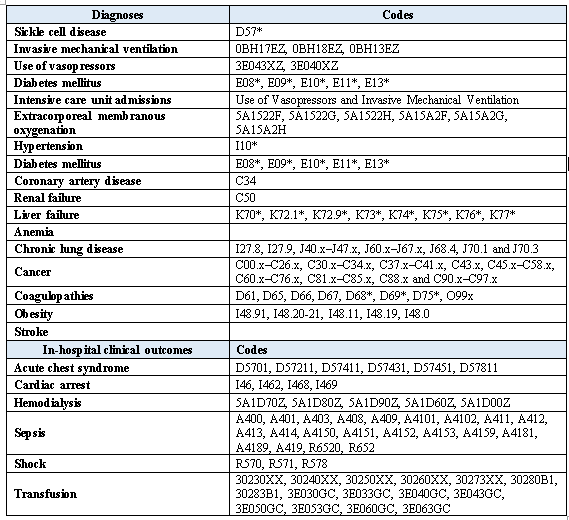

Supplement: Supplementary file 1 — Figure S1: ICD‐10 Codes Used to Identify Independent and Dependent Variables. Table S1: International Classification of Disease 10th Revision (ICD‐10) codes for comorbidities [file JHA2-6-e70111-s001.docx]
